# Do Probiotics Prevent Clostridium difficile-Associated Diarrhea?

**DOI:** 10.7759/cureus.27624

**Published:** 2022-08-02

**Authors:** Afrah Al Sharaby, Tahani M Abugoukh, Wefag Ahmed, Samah Ahmed, Abeer O Elshaikh

**Affiliations:** 1 Internal Medicine, Michigan State University, San Francisco, USA; 2 Medicine, Shendi University, Cedar Rapids, USA; 3 Pediatric Infectious Diseases, Sunydown State Medical Center, New York City, USA; 4 Internal Medicine, Michigan State University, Saskatoon, CAN; 5 Internal Medicine/Family Medicine, California Institute of Behavioral Neurosciences & Psychology, Fairfield, USA

**Keywords:** probiotics, prevention, clostridium difficile, diarrhea, efficacy

## Abstract

Clostridium difficile is a bacterium that affects the gastrointestinal tract and is the leading cause of antibiotic-associated diarrhea. A wide range of probiotics has been studied and used to prevent or treat Clostridium difficile-associated diarrhea (CDAD). Probiotics are microorganisms with unique characteristics that suppress dangerous gut bacteria through several mechanisms. The main objective of this study is to evaluate the efficacy and safety of probiotics in the prevention of CDAD. In this literature review, we searched PubMed and Google Scholar databases to gather related articles depending on predetermined eligibility criteria and found 13 papers of different study designs. We found that probiotics have promising effects in preventing CDAD. Additionally, they were safe and well-tolerated. Further randomized clinical trials with larger sample sizes and various patient groups are needed to better understand the advantages of probiotics and recommend the best dose and duration of probiotic treatment.

## Introduction and background

**Figure 1:**
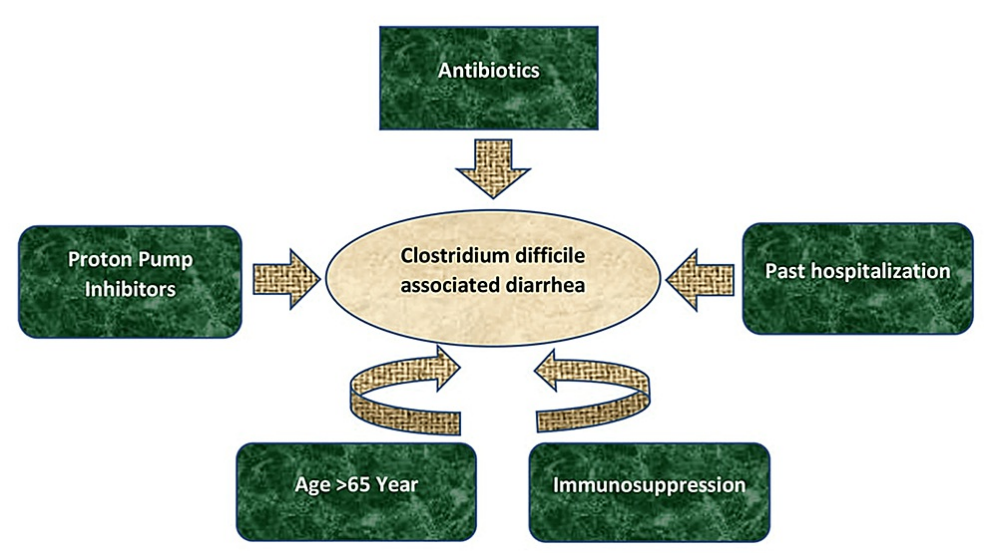
Clostridium difficile-associated diarrhea risk factors The figure is an original illustration created by one of the co-authors (Abeer Elshaikh) using Microsoft Word.

Clostridium difficile (CD) is a Gram-positive, spore, and toxin-forming bacteria that can affect the gastrointestinal (GI) tract [1]. The bacterium was first identified in 1977, and since then, it has been the most common cause of antibiotic-associated diarrhea (AAD) and colitis worldwide [2]. Clostridium difficile-associated diarrhea (CDAD) is caused by the effects of two exotoxins (toxin A and toxin B) produced by various pathogenic strains of CD, resulting in over 400,000 infections and nearly 29,000 fatalities per year in the United States alone [3,4]. Antibiotic use is a key risk factor for CD infection (CDI) [5]. It leads to the loss of gut microbial communities that protect against disease, allowing the organism to germinate and grow vegetatively when it enters the guts of vulnerable persons [5]. Other risk factors include age over 65, past hospitalization, immunosuppression, and proton pump inhibitors [6]. These risk factors are illustrated in Figure 1.

Probiotics, which include bacteria and yeast, are microbes that have been shown to have a role in preventing many diseases [7]. Probiotic microorganisms have recently received much attention, and their use is being explored as a viable supplemental treatment for various intestinal illnesses [7]. Species of the genera Lactobacillus, Bifidobacterium, and Enterococcus are among the numerous intestinal microorganisms that are thought to benefit the host by enhancing the gut microbial balance [8]. Probiotics can also include microorganisms that do not ordinarily live in the intestine, like Lactobacillus bulgaricus, Streptococcus thermophilus, and other bacteria commonly employed as starters in dairy products [8]. Probiotics have been suggested to prevent and treat CDAD [9].

This literature review aims to explore the efficacy of probiotics in preventing CDAD. We searched PubMed and Google Scholar to gather the related studies. We included free full-text articles written in English and published between January 1, 2012, and December 31, 2022. We had several types of studies as randomized control trials and systematic reviews. We excluded non-English studies that were published before 2012.

## Review

Efficacy of probiotics in the prevention of CDAD

Goldenberg et al. conducted a systematic review and meta‐analysis of 31 randomized controlled trials (RCTs), including 8,672 patients. They found evidence of moderate certainty suggests that probiotics are effective in preventing CDAD (number needed to benefit [NNTB] = 42 patients, 95% confidence interval [CI] 32-58) [9]. However, a post hoc subgroup analysis to investigate heterogeneity revealed that probiotics are effective in trials with a CDAD baseline risk greater than 5% (NNTB = 12; moderate certainty evidence) but not in trials with a risk less than 5% (low to moderate certainty evidence) [9].

Lau and Chamberlain assessed 26 RCTs comprising 7,957 patients in a systematic review and meta-analysis [10]. They found that the usage of probiotics decreased the incidence of CDAD by 60.5 % (relative risk [RR] =0.395; 95 % CI, 0.294-0.531; P=0.001) [10]. Probiotics were found to be effective in decreasing CDAD in both adults and children (59.5% and 65.9%, respectively), particularly among hospitalized patients [10]. Lactobacillus, Saccharomyces, and a combination of probiotics helped reduce the risk of CDAD (63.7%, 58.5%, and 58.2% reduction) [10].

In a systematic review and meta‐analysis of 16 RCTs of adult inpatients receiving antibiotics, there was a lower risk of AAD (RR 0.61, 95% CI 0.47-0.79) and CDI (RR 0.37, 95% CI 0.22-0.61) in patients randomly assigned to probiotic co-administration [11]. For AAD, the number needed to treat (NNT) was 11 (95 % CI 8-20), and for CDI, it was 14 (95% CI 9-50) [11]. The literature does not clearly indicate a preferred choice of probiotics, but some studies mentioned specific types of probiotics [11].

A study by Mills et al. reviewed recent clinical evidence and new approaches to probiotic development, which showed that traditional single and small combination probiotic agents demonstrated modest success in lowering the risk of CDI in high-risk patients receiving systemic antibiotics [12].

Similarly, Rodriguez and Miller conducted a systematic review with a meta-regression analysis of 19 RCTs; with 6,261 participants, including 3,277 patients in the probiotic group and 2,984 patients in the control group [13]. The studies suggest that probiotics reduce the incidence of CDI among high-risk individuals [13].

Furthermore, Alberda et al. performed a randomized clinical trial; the study included 32 patients from a single Intensive Care Unit (ICU) who were matched for age, body mass index (BMI), and illness severity, with the study goal of gathering a convenience sample of ICU patients who had started antibiotic medication [14]. They conclude that probiotic-containing drinks can be provided safely through a feeding tube and should be considered as a preventive measure for AAD and CDI in the ICU [14].

McFarland conducted meta-analyses that included a thorough search of both established and new literature databases [15]. It showed that four species of probiotic strains helped prevent primary CDI cases (S. boulardii, L. casei DN114001, and the mixture of Lactobacillus acidophilus and Bifido) [15]. Only two varieties of probiotics (S. boulardii and L. rhamnosus GG) had enough trials to assess secondary prevention of CDI [15].

In contrast, Allen et al. performed a randomized, double-blind, placebo-controlled, multicenter trial of inpatients aged 65 years and older who were exposed to one or more oral or parenteral antibiotics [16]. Of the 17,420 patients evaluated, 1,493 were randomly assigned to receive the microbial preparation, while 1,488 received a placebo [16]. AAD (including CDD) occurred in 159 (108% of the microbial preparation group and 153 (104% of the placebo group) (relative risk [RR] 104; 95% CI 084-128; p=071) [16]. They found no evidence that a multistrain preparation of lactobacilli and bifidobacteria was effective in AAD or CDD prevention [16].

Mechanism of action of probiotics

Chiu et al., in their study, reported that fecal microbiota transplantation's (FMT) mechanisms for effectively preventing or treating CDIs by modulating microbiota had been proposed to be through the influence on the metabolism of certain bile acids that affect C. difficile germination or vegetative growth [17].

Rodriguez and Miller conducted a systematic review with a meta-regression analysis of 19 RCTs; there were 6,261 participants, including 3,277 patients in the probiotic group and 2,984 patients in the control group [13]. They mentioned that certain probiotics could counteract C. difficile (CD) toxins A and B [13]. Probiotics have been shown to have protective effects in vitro and in preclinical experiments [13]. Lactobacillus lactis, for example, generates a lytic peptide that inhibits CD toxin action, while S. boulardii produces a protease that inhibits the activity of CD toxin [13].

According to Zhu et al., butyrate-producing bacteria (probiotics containing bacteria that produce butyrate) can ferment undigested carbohydrates in the intestine lumen, resulting in acidifying short-chain fatty acids (SCFAs) like butyric acid [18]. Moreover, Butyrate is a powerful histone deacetylase inhibitor that promotes the proliferation and activation of regulatory T-cells (Treg cells) and plays a key role in immunological control [18]. By regulating the activity of G protein-coupled receptors, NF-B, JAK/STAT, and other inflammation-related pathways, microbiota-derived Butyrate can limit the release of pro-inflammatory cytokines, reducing intestinal inflammation and preserving intestinal immunological homeostasis [18].

Wong et al. conducted a randomized control trial on people aged 18 and up, of any race or gender, who had been diagnosed with a spinal cord injury [19]. The research explained how probiotics operate [19]. Probiotics that colonize the GI tract are efficient probiotics that work by preventing potentially dangerous bacteria from colonizing the gut [19]. Bacteriocins are antimicrobial chemicals produced by Lactobacillus strains that may suppress infections such as Bacillus, Staphylococcus, and Enterococcus species [19]. L. acidophilus produces a bacteriocin that has been demonstrated to inhibit Listeria innocua and Listeria monocytogenes [19].

Stier and Bischoff conducted a systematic review and concluded that the probiotic S. boulardii reduces pathogen adhesion or colonization and diminishes the overreacting inflammatory immune response [20]. As a result, the integrity of the intestinal epithelial cell layer is retained or restored, and fluid leakage into the intestinal lumen is reduced [20].

Yun et al., in an experimental study, revealed that L. acidophilus has a bactericidal effect on C. difficile in addition to mRNA downregulation of C. difficile virulence genes [21]. L. acidophilus also inhibits C. difficile growth in the Clostridium difficile infected (CDI) mouse model, which may be due to a lower pH caused by organic acids produced by the probiotic bacterium [21].

Safety of probiotics

The safety of probiotics is supported by Goldenberg et al. based on their systematic review and meta-analysis; adverse effects were reported in 32 included trials. However, patients in the control groups experienced more adverse events [9].

Additional support for the safety of probiotics can be found in the systematic review and meta‐analysis of 16 RCTs by Pattani et al., which involves adult inpatients. In these RCTs, no life-threatening adverse effects of probiotics were reported [11]. Despite case reports of toxic effects in patients with exceptional cases, probiotics had an excellent safety profile, with most side effects being gastrointestinal upset [11].

Rodriguez and Miller conducted a systematic review with a meta-regression analysis of 19 RCTs. These studies mentioned that, like other vitamins or medications, probiotics might have negative side effects [13]. Cramping, flatulence, nausea, fever, soft stools and taste disturbance were the most prevalent symptoms [13]. They found that adverse events occurred in 15.9% and 14.2% of the control and probiotic groups, respectively. It is also worth noting that no incidences of bacteremia or fungemia were reported [13].

In a systematic review, Stier and Bischoff reported that the probiotic S. boulardii consumption is considered safe in all age groups except severely ill patients, where the risk of developing fungemia is estimated to be one in every 5.6 million users [20]. The characteristics of included studies are summarized in Table 1.

**Table 1:** Summary of the characteristics of included studies RCTs = randomized controlled trials, CDAD = Clostridium difficile-associated diarrhea, AAD = antibiotic-associated diarrhea, CDD = C. difficile diarrhea, CDI = Clostridium difficile infection, CD = Clostridium difficile.

Author	Type of Study	Participants	Findings
Goldenberg et al. [9]	A systematic review, including 31 RCTs	8672 adult (> 18 years) and pediatric patients (0 to 18 years of age) receiving antibiotic therapy for any reason.	Probiotics are helpful at preventing CDAD, according to evidence they discovered, which is moderately certain.
Lau and Chamberlain [10]	systematic review and meta-analysis, including 26 RCTs	7,957 patients	Probiotic use was observed to reduce the incidence of CDAD by 60.5% ([RR] =0.395; 95 percent [CI], 0.294-0.531; P=0.001). Probiotics were found to be particularly helpful for hospitalized patients in reducing CDAD in adults and children (59.5% and 65.9%, respectively).
Pattani et al. [11]	systematic review and meta-analysis, including 16 RCTs	adult inpatients, the range of mean ages for patients randomly assigned to probiotic was 33–79.9 years and to placebo was 33–78.5 years	There was a lower risk of AAD (RR 0.61, 95% CI 0.47 - 0.79) and CDI (RR 0.37, 95% CI 0.22 - 0.61) in patients randomly assigned to probiotic co-administration.
Mills et al. [12]	Review		probiotic agents demonstrated modest success in lowering the risk of CDI in high-risk patients receiving systemic antibiotics.
Rodriguez & Miller [13]	systematic review with meta-regression analysis, including 19 RCTs	14,933 patients	The studies show the benefit of probiotics in high-risk groups with a reduction in the incidence of CD. Specific probiotics have the capability to antagonize CD toxins A and B.
Alberda et al. [14]	RCT	32 patients from a single ICU	A probiotic containing drink can safely be delivered via a feeding tube and should be considered a preventative measure for AAD and CDI in ICU.
McFarland [15]	a meta-analysis, including 25 RCTs	4476 participants, pediatric or adult populations (inpatient or outpatients).	It revealed that four species of probiotic strains helped prevent primary CDI cases. Only two varieties of probiotics had enough trials to assess secondary prevention of CDI.
Allen et al. [16]	RCT	2981 inpatients, aged 65 years and older	There is no proof that a multistrain mixture of bifidobacteria and lactobacilli prevents AAD or CDD.
Chiu et al. [17]	Review		Microbiota transplantation's (FMT) mechanisms, effectively prevent or treat CDIs through the influence on the metabolism of certain bile acids that affect C. difficile germination or vegetative growth.
Zhu et al. [18]	review		By regulating the activity of G protein-coupled receptors, NF-B, JAK/STAT, and other inflammation-related pathways, microbiota-derived Butyrate can limit the release of pro-inflammatory cytokines, reducing intestinal inflammation and preserving intestinal immunological homeostasis.
Wong et al. [19]	Meta-analysis and systematic review	people aged 18 and up, of any race or gender, who had been diagnosed with a spinal cord injury	It explained how probiotics operate to inhibit harmful bacteria through producing bacteriocin.
Stier and Bischoff [20]	Review		the probiotic Saccharomyces boulardii reduces pathogen adhesion or colonization and diminishes the overreacting inflammatory immune response.
Yun et al. [21]	experimental study		Lactobacillus acidophilus (L. acidophilus) has a bactericidal effect on C. difficile in addition to mRNA downregulation of C. difficile virulence genes. L. acidophilus also inhibits C. difficile growth in the CDI mouse model.

Limitations

Although this review contains several randomized control trials and meta-analyses, it still has certain limitations. In this review, we only looked at publications published in English between 2012 and 2022 and had a full text available for free. Additionally, some studies have a small sample size.

## Conclusions

We studied the effectiveness of probiotics in preventing diarrhea caused by CD. All included studies showed significant benefits of probiotics in preventing and treating CDAD. However, one study failed to demonstrate the beneficial effect of probiotics. The articles demonstrated that probiotics work primarily by regulating the microbiota and inhibiting Clostridium difficile virulence and proliferation. Additionally, the studies revealed that the probiotics were well-tolerated by all study participants, and no major adverse effects were observed except for mild gastrointestinal upsets. Thus, it is worth considering probiotics as adjuvant therapy in all patients at risk of developing CDAD to optimize disease prevention and subsequent complications. More randomized clinical trials that include larger sample sizes and diverse patient populations are needed to further explore the benefits of probiotics and advise the optimal dose and duration of probiotic treatment.
